# Multifunctional PBAT/Curcumin Bioactive Composite Films with Colorimetric Properties for Packaging

**DOI:** 10.3390/polym18162004

**Published:** 2026-08-17

**Authors:** Yujie Guo, Hong Yu, Shunlin Yang, Yanziwen Zhang, Xiucheng Zhao, Lihua Zhang, Haibo Xie

**Affiliations:** 1 Department of Polymer Materials and Engineering, College of Materials and Metallurgy, Guizhou University, Guiyang 550025, China; gyj714006@126.com (Y.G.); yh95272026@163.com (H.Y.); yang17882659368@163.com (S.Y.); zyzw17371725205@163.com (Y.Z.); zxcol1018319@163.com (X.Z.); 2 Technology Innovation Center for High-Efficiency Utilization of Bamboo-Based Biomass in Guizhou Province, Guiyang 550025, China

**Keywords:** PBAT, curcumin, multifunctional properties, active and intelligent packaging

## Abstract

The extensive use of common petroleum-based plastics in food packaging has raised serious environmental concerns, accelerating the search for biodegradable and functional alternatives. In this study, poly(butylene adipate-co-terephthalate)/curcumin (PBAT/Cur) bioactive composite films with colorimetric sensing properties were successfully prepared via solution casting. A systematic characterization was conducted on the structural, morphological, barrier, antioxidant, antibacterial, and colorimetric properties of the films. The optimal PBAT/Cur1% films exhibited potent antioxidant activity (DPPH scavenging up to 95.6%) and moderate antibacterial activity against *E. coli* and *S. aureus*. Additionally, curcumin incorporation not only increased the water contact angle of the PBAT/Cur1% films, indicating enhanced surface hydrophobicity, but also concurrently improved the barrier properties, as evidenced by a reduced water vapor permeability (WVP of 14.58 g·mm/m^2^·day·kPa) and a lower oxygen transmission rate (OTR of 7.533 × 10^−3^ cm^3^/m^2^·day·Pa) compared to the neat PBAT films. Notably, the films displayed a distinct and rapid color change from yellow to reddish-brown upon exposure to ammonia vapor, suggesting their promise for on-package visual freshness indication. These findings highlight PBAT/Cur composite films as a sustainable option for active and intelligent food packaging, with combined antioxidant, antibacterial, and colorimetric properties, making them promising for packaging protein-rich foods (e.g., meat and seafood).

## 1. Introduction

Petroleum-based plastics have been widely used in food packaging for decades, owing to their low cost, mechanical robustness, easy processability, and high barrier performance. However, their persistence in the environment has led to serious pollution, including plastic waste accumulation in landfills and oceans, and the generation of harmful microplastics [1,2]. In response to global plastic restriction policies and growing public demand for sustainable food preservation, biodegradable polymers have shown great promise as substitutes for conventional petroleum-based packaging materials [3,4]. Among these, poly(butylene adipate-co-terephthalate) (PBAT) has garnered significant interest in food packaging [5,6,7]. As a biodegradable aliphatic-aromatic copolyester, PBAT combines the flexibility of aliphatic chains with the thermal/mechanical resilience of aromatic segments, featuring excellent film-forming ability and good processability [8]. Compared with other widely studied biodegradable polymers such as brittle PLA, PBAT exhibits favorable ductility for flexible film applications. From an economic perspective, PBAT is more cost-effective than PHA and competitive with PLA, and its price gap with conventional polyolefins has been narrowing with the rapid expansion of global production capacity. PBAT can undergo complete biodegradation under industrial composting conditions, offering a sustainable end-of-life pathway that aligns with circular economy principles and alleviates the environmental burden of plastic waste [9,10].

Food spoilage remains a critical global concern, with approximately one-third of all food produced for human consumption lost or wasted annually, largely due to microbial contamination and oxidative degradation during storage and distribution. Despite the above-mentioned advantages, neat PBAT films lack the bioactive functionalities to extend food shelf life during packaging, where bacterial contamination and oxidative degradation remain major challenges leading to food spoilage and waste [11,12]. To overcome this limitation, various strategies have been explored for active food packaging. One widely adopted approach involves the incorporation of active inorganic nanoparticles, such as silver [13,14,15], zinc oxide [11,16], and titanium dioxide [17,18] as fillers to impart broad-spectrum antimicrobial activities. Alternatively, some natural agents, including essential oils (e.g., thymol [19], carvacrol [20], cinnamon [21,22]), plant extracts (e.g., tea polyphenols [23], grape seed extract [24]), and organic acids (e.g., gallic acid [25]), have been explored to impart antimicrobial and antioxidant functions to PBAT-based packaging films. Among these, curcumin (Cur), a GRAS-certified polyphenolic compound derived from turmeric (*Curcuma longa*), has gained considerable interest as a multifunctional food packaging additive [26,27,28,29]. As an antioxidant, curcumin contains phenolic hydroxyl groups that effectively scavenge free radicals, thereby retarding oxidative deterioration of packaged foods [30]. As an antibacterial agent, curcumin exhibits broad-spectrum activity against food-borne pathogens such as *E. coli* and *S. aureus* via cell membrane disruption and inhibition of proliferation, thus imparting effective antibacterial activities [31].

Beyond its active functions as an antioxidant and antibacterial agent, curcumin also possesses unique pH-responsive colorimetric functionality that enables its application in intelligent food packaging. In acidic or neutral environments, curcumin exhibits a characteristic yellow color; however, under alkaline conditions, which typically result from the pH increase caused by the accumulation of volatile amines during protein-rich food spoilage, it changes from yellow to reddish-brown [27,32]. This color change arises from the molecular structure of curcumin. Its phenolic hydroxyl groups and β-diketone moiety form a conjugated system that gives a yellow color under acidic/neutral conditions, while upon alkaline exposure, deprotonation extends the conjugation, shifting the color to reddish-brown [26]. Therefore, curcumin can serve as an effective colorimetric indicator for real-time visual freshness assessment, enabling consumers to evaluate packaged food quality (especially meat, fish, and dairy) without opening the package [33,34,35]. For instance, Choi et al. developed a biodegradable PHB/curcumin composite film that gradually changed from yellow to pale red in response to volatile alkaline nitrogen compounds during meat and seafood spoilage, demonstrating its practical use as an intelligent freshness indicator [36]. Although Roy and Rhim demonstrated that curcumin-incorporated PBAT films exhibited antioxidant activity and modest antibacterial activity [37], their work primarily focused on the active packaging functions without exploring the colorimetric sensing behavior of curcumin within the PBAT matrix.

Thus, in this study, PBAT/curcumin (PBAT/Cur) bioactive composite films were developed with integrated antioxidant, antibacterial, and pH-responsive colorimetric functionalities, suggesting their potential applicability in dual-purpose packaging systems. The structural, mechanical and barrier properties of the films were characterized. The antioxidant and antibacterial activities were determined, and the colorimetric response was monitored upon exposure to ammonia vapor. This study provides a proof-of-concept demonstration that curcumin can serve as a multi-functional additive within a PBAT matrix, combining active preservation with ammonia-sensing capability.

## 2. Materials and Methods

### 2.1. Materials

PBAT (TH801T) was supplied by Xinjiang Blue Ridge Tunhe Co., Ltd. (Urumqi, China). Curcumin (≥98%) and DPPH were obtained from Aladdin Reagent Co., Ltd. (Shanghai, China). ABTS was provided by Yuanye Bio-Technology Co., Ltd. (Shanghai, China). Chloroform was acquired from Chuandong Chemical Co., Ltd. (Chongqing, China). All reagents were used as received.

### 2.2. Fabrication of PBAT/Cur Composite Films

**Figure 1:**
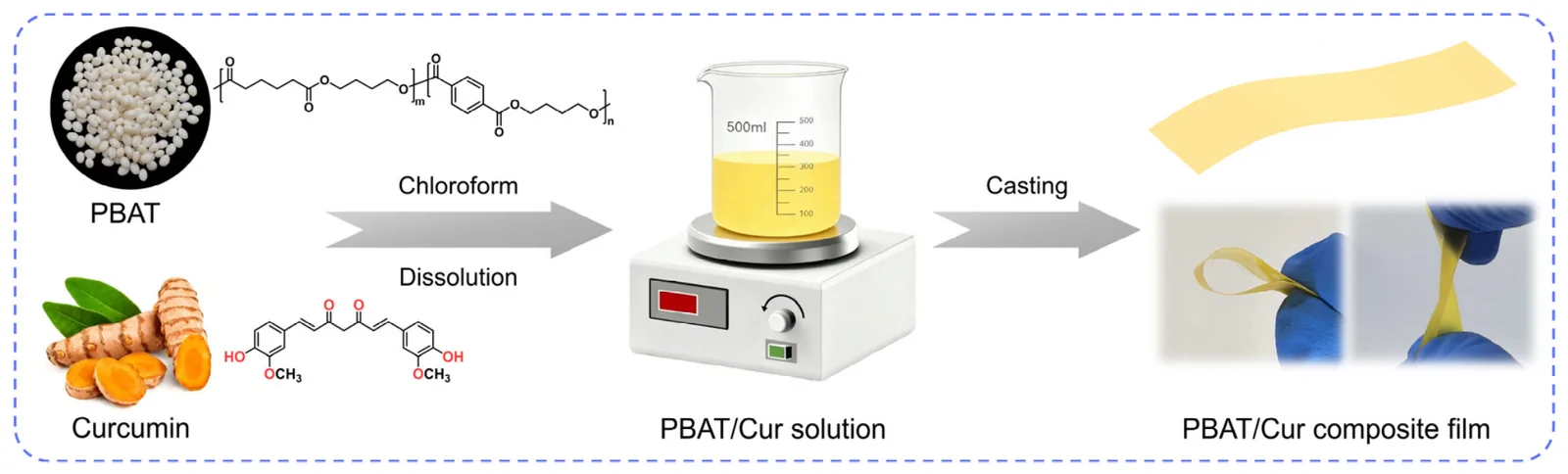
Schematic illustration of the fabrication procedure for PBAT/Cur composite films.

The fabrication procedure of the PBAT/Cur composite films is depicted in Figure 1. The films were fabricated through a solution casting method [25,38]. A homogeneous solution of PBAT (1.6 g) and curcumin (0.2, 0.5, 1.0, 2.0 wt% relative to PBAT) in 40 mL of chloroform was prepared with stirring at 25 ± 2 °C for 2 h. The casting solution was poured into glass Petri dishes (150 mm diameter) and the solvent was evaporated in a fume hood at 25 ± 2 °C for 24 h (relative humidity: 45 ± 5%). A neat PBAT film without curcumin was also prepared as a control. The PBAT/Cur composite films were denoted as PBAT/Cur0.2%, PBAT/Cur0.5%, PBAT/Cur1%, and PBAT/Cur2%, according to the curcumin content.

### 2.3. Basic Characterization

Fourier transform infrared (FTIR) spectra were recorded on a Thermo Scientific Nicolet iS20 spectrometer (Thermo Fisher Scientific, Waltham, MA, USA). Surface morphologies were examined using a field-emission scanning electron microscope (FE-SEM, GeminiSEM 300, ZEISS, Oberkochen, Germany). X-ray diffraction (XRD) patterns were collected using a Rigaku Ultima IV diffractometer (Rigaku Corporation, Akishima, Japan) with Cu Kα radiation (λ = 1.5406 Å) at 45 kV and 200 mA over a 2θ range of 5–90°. Thermogravimetric analysis (TGA) was performed on a Netzsch TG209F1 Libra instrument (Netzsch, Selb, Germany) under a nitrogen atmosphere, with samples heated from 30 °C to 800 °C at a heating rate of 10 °C/min. Water contact angle (WCA) measurements were carried out using a WDW-100 contact angle system (Shanghai Hualong Test Instruments, Shanghai, China). The tensile behavior of the films was characterized using a universal testing machine (CMT6103, MTS Industrial System, Shanghai, China) at a tensile speed of 10 mm/min. Prior to testing, film specimens were cut into rectangular strips with dimensions of 60 mm × 10 mm. Differential scanning calorimetry (DSC) was carried out using a TA Q2000 apparatus (TA Instruments, New Castle, DE, USA). To eliminate thermal history, all samples were first heated to 200 °C, cooled to −50 °C, and then reheated to 190 °C at a heating rate of 10 °C/min. The second heating thermograms were recorded for analysis.

### 2.4. Film Color Parameters

The color parameters of the films were measured using a 3nh CR8 colorimeter (Sanenshi, China) using a standard white plate (L = 90.33, a = −0.35, b = −6.58). The L* (lightness), a* (red/green), and b* (yellow/blue) values were recorded, and the total color difference (ΔE) was calculated according to the following equation:

(1) $$\Delta E\;=\;\sqrt{{\left(L*-L\right)}^{2}+{\left(a*-a\right)}^{2}+{\left(b*-b\right)}^{2}}$$

### 2.5. Barrier Properties

The water vapor permeability (WVP) was measured using a W3/031 permeability tester (Labthink, Jinan, China) at 23 °C and 90% RH. The oxygen transmission rate (OTR) was determined with a VAC-V2 differential pressure gas permeability tester (Labthink, Jinan, China) at 23 °C and 50% RH. The grease resistance was evaluated using the Kit test, following TAPPI T559 cm-12 standard [39]. In this method, samples were exposed to 12 test solutions with various formulations of castor oil, n-heptane, and toluene. Solution 1 consisted of pure castor oil, while Solution 12 comprised 45% toluene and 55% n-heptane. The Kit number was defined as the highest solution number that did not cause staining on the paper surface, with a higher number indicating better grease resistance.

### 2.6. Antioxidant Activity

The antioxidant activity was assessed by DPPH and ABTS assays [40,41]. For DPPH, film specimens (50 mg) were incubated in 5 mL of 0.1 mM DPPH ethanol solution at room temperature for 30 min in the dark. The absorbance of the supernatant was then measured at 517 nm using a UV-vis spectrophotometer (Unico UV2800A, Shanghai, China).

For the ABTS assay, a 7 mM ABTS solution was mixed with a 2.4 mM potassium persulfate solution (2:1, *v*/*v*) and kept in the dark for 12 h. The resulting solution was diluted with water to an absorbance of 0.7 at 734 nm. Films (50 mg) were incubated in 5 mL of the diluted ABTS solution at room temperature for 50 min in the dark, after which absorbance at 734 nm was recorded.

The antioxidant activity was calculated as follows:

Antioxidant activity (%) = (A_0_ − A_1_)/A_0_ × 100 (2)

where A_0_ represents the absorbance of the control (DPPH or ABTS solution without sample), and A_1_ denotes the absorbance after incubation with the sample.

### 2.7. Antibacterial Activity Assay

The antibacterial activity of the films was tested against Gram-negative *E. coli* (ATCC 25922) and Gram-positive *S. aureus* (ATCC 6538), using the colony counting method [40,42]. Each sterilized film sample (1 × 1 cm) was immersed in 10 mL of nutrient broth and inoculated with 100 μL of bacterial suspension (~10^5^ CFU/mL) of *E. coli* or *S. aureus*. A blank control without any sample was also prepared. After incubation at 37 °C for 18 h with shaking at 180 rpm, the bacterial suspensions were serially diluted, and 100 µL aliquots of appropriate dilutions were spread onto nutrient agar plates. Each experiment was performed in triplicate. The plates were incubated at 37 °C overnight, and the viable colonies were counted. The antibacterial rate was calculated as:

Antibacterial rate (%) = (*N*_control_ − *N*_sample_)/*N*_control_ × 100% (3)

where *N*_control_ and *N*_sample_ represent the numbers of colonies in the control and sample groups, respectively.

### 2.8. Curcumin Release

The release behavior of curcumin from the PBAT/Cur1% film was investigated in two release media, namely deionized water and 90% ethanol (*v*/*v*), which were selected as aqueous and fatty food simulants, respectively. Film samples with dimensions of 1.5 cm × 1.5 cm were immersed in 20 mL of each release medium in sealed glass vials. The concentration of released curcumin was determined by UV-vis spectrophotometry at the characteristic absorption wavelength of curcumin (428 nm) using a pre-established calibration curve. All release experiments were conducted in triplicate.

### 2.9. Enzymatic Degradation

The enzymatic degradation was evaluated using lipase (Yuanye Bio-Technology Co., Ltd., Shanghai, China) in phosphate-buffered saline (PBS). Film samples were cut into rectangular pieces of 1.5 mm × 1.5 mm and placed in separate glass vials containing 12 mL of PBS (pH 7.20 ± 0.01) with lipase at a concentration of 6 mg/mL. The vials were incubated in a constant-temperature shaking incubator at 45 °C. At predetermined time intervals (0, 4, 8, 12, 16, and 20 days), the samples were retrieved, rinsed with deionized water to remove residual lipase, gently wiped with tissue paper, and dried under vacuum at 50 °C to constant weight. The weight loss of each sample was calculated to evaluate the degradation rate.

### 2.10. Colorimetric Analysis

To investigate the pH-responsive color-changing behavior of curcumin, a curcumin stock solution at a concentration of 33.3 μg/mL was firstly prepared by dissolving 1 mg curcumin in 30 mL anhydrous ethanol. Aqueous solutions with pH values ranging from 3 to 11 were obtained by diluting hydrochloric acid and sodium hydroxide aqueous solutions, and then mixed with the curcumin stock solution at a volume ratio of 7:3 (*v*/*v*). The absorption spectra of the resulting solutions were measured using a Unico UV2800A UV-vis spectrophotometer.

To evaluate the ammonia-responsive colorimetric response of the PBAT/Cur films, aqueous ammonia solutions with concentrations of 5 wt%, 10 wt%, and 25 wt% were prepared and transferred into separate 20 mL glass vials. The PBAT/Cur film was placed over the mouth of the glass vial containing the ammonia solution and fixed with a rubber band. The distance between the film and the liquid surface was maintained at 3 cm. The vial was kept at 25 °C during exposure. Digital photographs were taken to document the visible color changes, and color parameters were recorded after exposure for 1, 5, and 30 min.

### 2.11. Statistical Analysis

All the experiments were conducted in triplicate and the data was expressed as means ± standard deviation. Statistical analysis was performed using SPSS software (version 20.0, SPSS Inc., Chicago, IL, USA). Data were analyzed by one-way analysis of variance (ANOVA), followed by Duncan’s multiple range test, with a significance level of *p* < 0.05.

## 3. Results and Discussion

### 3.1. Visual Appearance, Color Parameters and Thickness

**Figure 2:**
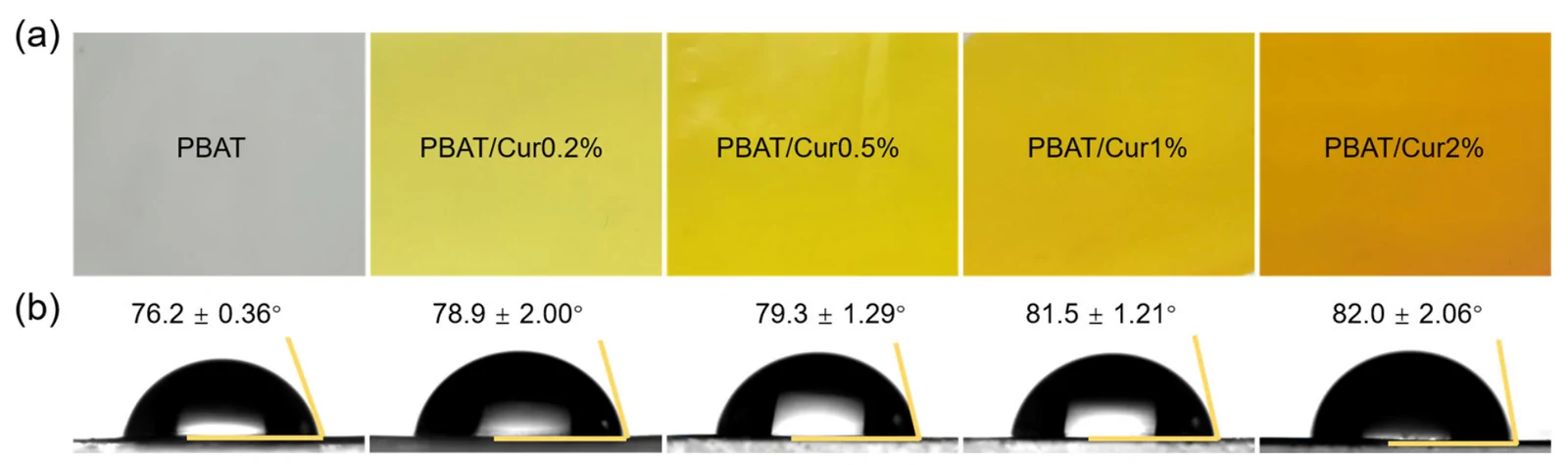
(**a**) Photographs and (**b**) WCA images of the neat PBAT and PBAT/Cur composite films. All values were presented as the mean ± SD (*n* = 3).

Figure 2a shows the photographs of the PBAT and PBAT/curcumin films. The addition of curcumin resulted in a progressive color change from translucent to bright yellow, while the PBAT/Cur2% film exhibited excessively deep coloration. The color parameters of these films are summarized in Table 1. The neat PBAT film exhibited a light translucent appearance with L*, a*, and b* values of 89.74, −0.47, and −4.61, respectively. Upon the incorporation of curcumin, the L* value (brightness) slightly decreased, while the a* value decreased significantly upon the addition of 0.2 wt% curcumin, and then increased progressively with further curcumin loading. The b* value (yellowness) increased markedly with increasing curcumin content, indicating a pronounced color change toward reddish-yellow. Consequently, the total color difference (ΔE) increased substantially from 2.06 for neat PBAT to 92.29 for the PBAT/Cur2% film, reflecting the strong coloring effect of curcumin (*p* < 0.05). These results suggest the successful incorporation of curcumin into the PBAT matrix and are consistent with the visual appearance of the films. Similar color changes have also been observed in other curcumin-incorporated polymer systems, such as PLA/Cur [43] and gelatin/Cur [44] composite films, where the yellowish coloration intensified with increasing curcumin loading.

Furthermore, the color stability was assessed by examining the color changes in the PBAT/Cur1% film after being stored at 25 °C for 60 days. The color parameters exhibited only minor changes: L* decreased slightly from 82.10 to 81.61, a* increased from 0.11 to 1.76, and b* increased from 81.27 to 83.05. The calculated total color difference (ΔE ≈ 2.48) was below 3, indicating that the color change is barely perceptible to the human eye. This excellent color stability is attributable to the protective effect of the PBAT matrix, which encapsulates curcumin and reduces its direct exposure to environmental factors such as light and oxygen.

The film thickness also varied with curcumin loading. As shown in Table 1, the average thickness increased progressively with curcumin content, ranging from 68 ± 1.5 μm (neat PBAT) to 95 ± 0.6 μm (PBAT/Cur2%), which is attributable to the increasing solid content upon curcumin incorporation.

### 3.2. Surface Hydrophobicity

The surface hydrophobicity of the PBAT/Cur composite films was evaluated by WCA measurements (Figure 2b). The neat PBAT film exhibited a contact angle of 76.2°, indicating a weakly hydrophobic surface, which is in general agreement with previous works [45]. Upon curcumin incorporation, the contact angle increased progressively with curcumin loading, reaching 81.5° at 1.0 wt% and 82.0° at 2.0 wt% curcumin. This enhancement is attributed to the hydrophobic nature of curcumin, creating a more non-polar surface layer that reduces the affinity for water molecules. Similar enhancement of surface hydrophobicity upon curcumin incorporation has been reported for other polymer systems, such as cellulose [46], PLA [43], and LDPE [30] composite films.

### 3.3. Antioxidant Activities

**Figure 3:**
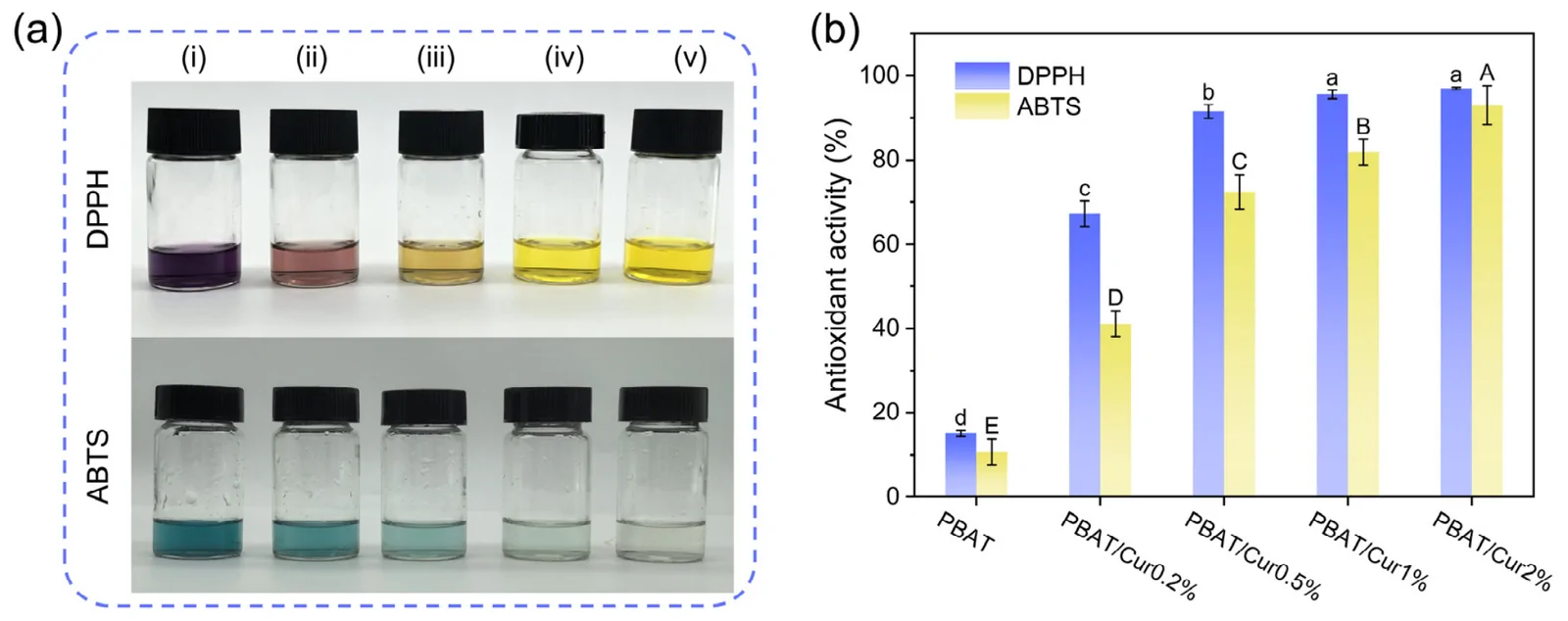
(**a**) Color changes of DPPH and ABTS solutions after incubation with (**i**) PBAT, (**ii**) PBAT/Cur0.2%, (**iii**) PBAT/Cur0.5%, (**iv**) PBAT/Cur1%, and (**v**) PBAT/Cur2%. (**b**) Quantitative radical scavenging efficacy of the neat PBAT and PBAT/Cur films. All values were presented as the mean ± SD (*n* = 3). Different lowercase letters (a–d) represent significant differences for DPPH assay; different uppercase letters (A–E) indicate significant differences for ABTS assay (*p* < 0.05).

The incorporation of natural antioxidants into polymer matrices is a promising strategy to impart active functions to food packaging. Curcumin, a polyphenolic compound, is known for its capacity to scavenge free radicals and inhibit oxidative reactions [26,47]. The antioxidant activity of the PBAT/curcumin films was evaluated using DPPH and ABTS assays, with the results presented in Figure 3a,b. The neat PBAT film showed limited antioxidant activity, with DPPH and ABTS scavenging rates of only 15.1% and 10.6%, respectively. With increasing curcumin content, the purple DPPH solution gradually faded to pale yellow, and the blue–green ABTS solution turned nearly colorless, indicating effective radical scavenging. These visual observations are consistent with the quantitative results. The DPPH radical scavenging activity of the PBAT/curcumin composite films reached 67.2% and 95.6% for the PBAT/Cur0.2% and PBAT/Cur1% films, respectively. Similarly, the ABTS scavenging activity exhibited a consistent upward trend, with values of 41.2% and 81.8% at the same curcumin contents. These results demonstrate a clear dose-dependent antioxidant response, attributed to the increasing phenolic hydroxyl groups with rising curcumin content. The clear trend between curcumin loading and radical scavenging efficiency indicates that curcumin retains its bioactive functionality within the PBAT matrix. Considering the above-mentioned variations in color, surface hydrophobicity and antioxidant performance, the 1 wt% curcumin loading was selected for further comprehensive characterizations in subsequent experiments.

The DPPH radical scavenging activity of the PBAT/Cur1% film was further evaluated after 60 days of storage at 25 °C. The initial activity was 95.6%, which decreased slightly to 91.56% after storage, representing a marginal loss. This minimal decline indicates that the embedded curcumin retains its radical-scavenging functionality effectively during medium-term ambient storage.

### 3.4. FTIR Analysis

**Figure 4:**
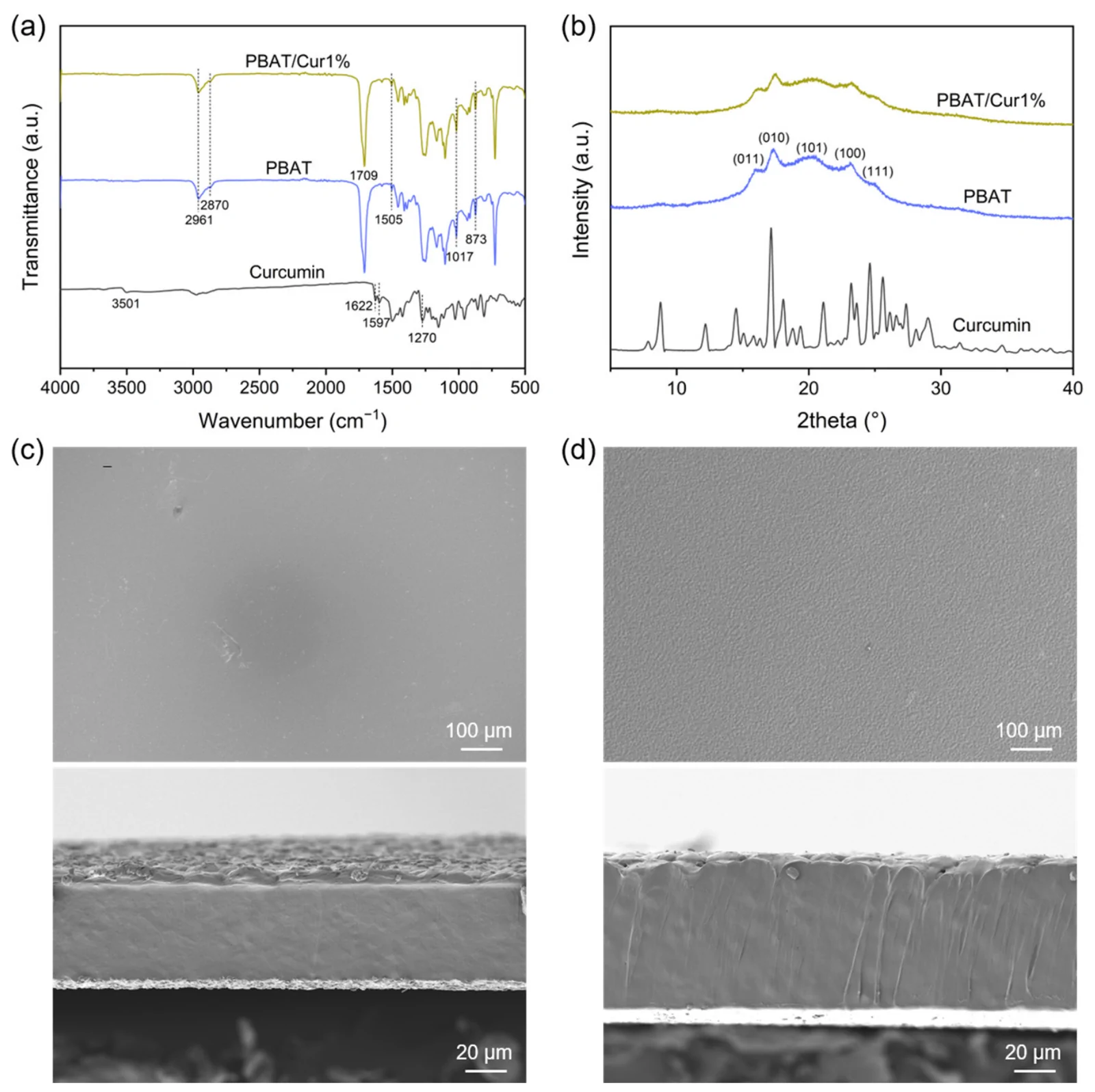
(**a**) FTIR spectra, (**b**) XRD patterns, and surface and cross-sectional SEM images of (**c**) neat PBAT and (**d**) PBAT/Cur1% films.

Figure 4a displays the FTIR spectra of neat PBAT and PBAT/Cur1% films. The PBAT spectrum displayed characteristic peaks at 2961 and 2870 cm^−1^, corresponding to the asymmetric and symmetric stretching of aliphatic CH_2_ groups. The strong absorption at 1709 cm^−1^ was attributed to the C=O stretching vibration of the ester linkages [48]. The peaks at 1505, 1017, and 873 cm^−1^ were assigned to the aromatic ring vibrations of the terephthalate units in PBAT. The characteristic bands of curcumin were observed at 3501 cm^−1^ (O–H), 1622 cm^−1^ (conjugated C=O stretching), 1597 cm^−1^ (aromatic C=C stretching), and 1270 cm^−1^ (C–O), confirming the presence of phenolic hydroxyl, carbonyl, and aromatic groups [31,36,49]. With the incorporation of curcumin, no new peaks appeared in the PBAT/Cur spectrum, indicating that no new chemical bonds or significant changes in functional groups were observed.

### 3.5. XRD Analysis

Figure 4b shows the XRD patterns of the PBAT-based films. The neat PBAT film exhibited a semi-crystalline structure, with characteristic diffraction peaks at 2θ = 16.0°, 17.3°, 20.4°, 23.2°, and 24.9°, corresponding to the (011), (010), (101), (100), and (111) crystal planes, respectively [50,51]. Curcumin showed multiple sharp and intense diffraction peaks, indicating its high crystallinity, which is in agreement with the literature results [46,52,53]. However, the characteristic peaks of crystalline curcumin were absent in the PBAT/Cur composite films, implying that curcumin was dispersed in an amorphous state within the PBAT matrix. Similar observations have also been reported for curcumin incorporated into other polymer matrices such as cellulose [46], chitosan [54], soy protein isolate [55], and PLA/PPC [56]. Meanwhile, the PBAT/Cur composite film exhibited similar diffraction peaks to neat PBAT, implying that the incorporation of curcumin did not alter the crystal structure of PBAT. Quantitative analysis revealed that the crystallinity decreased from 9.78% (neat PBAT) to 6.87% (PBAT/Cur1%), probably due to the disruption effect of curcumin on the crystalline domains of PBAT.

### 3.6. SEM

The surface and cross-sectional morphology of the neat PBAT and PBAT/Cur1% composite films were further examined by SEM (Figure 4c,d). The pristine PBAT film exhibited a smooth, homogeneous, and defect-free surface, indicating its good film-forming ability. Upon curcumin incorporation, the PBAT/Cur1% film retained a relatively smooth surface with no obvious aggregates, suggesting a fairly good dispersion of curcumin within the polymer matrix. Cross-sectional SEM images further confirmed this observation.

### 3.7. Mechanical and Thermal Properties

**Figure 5:**
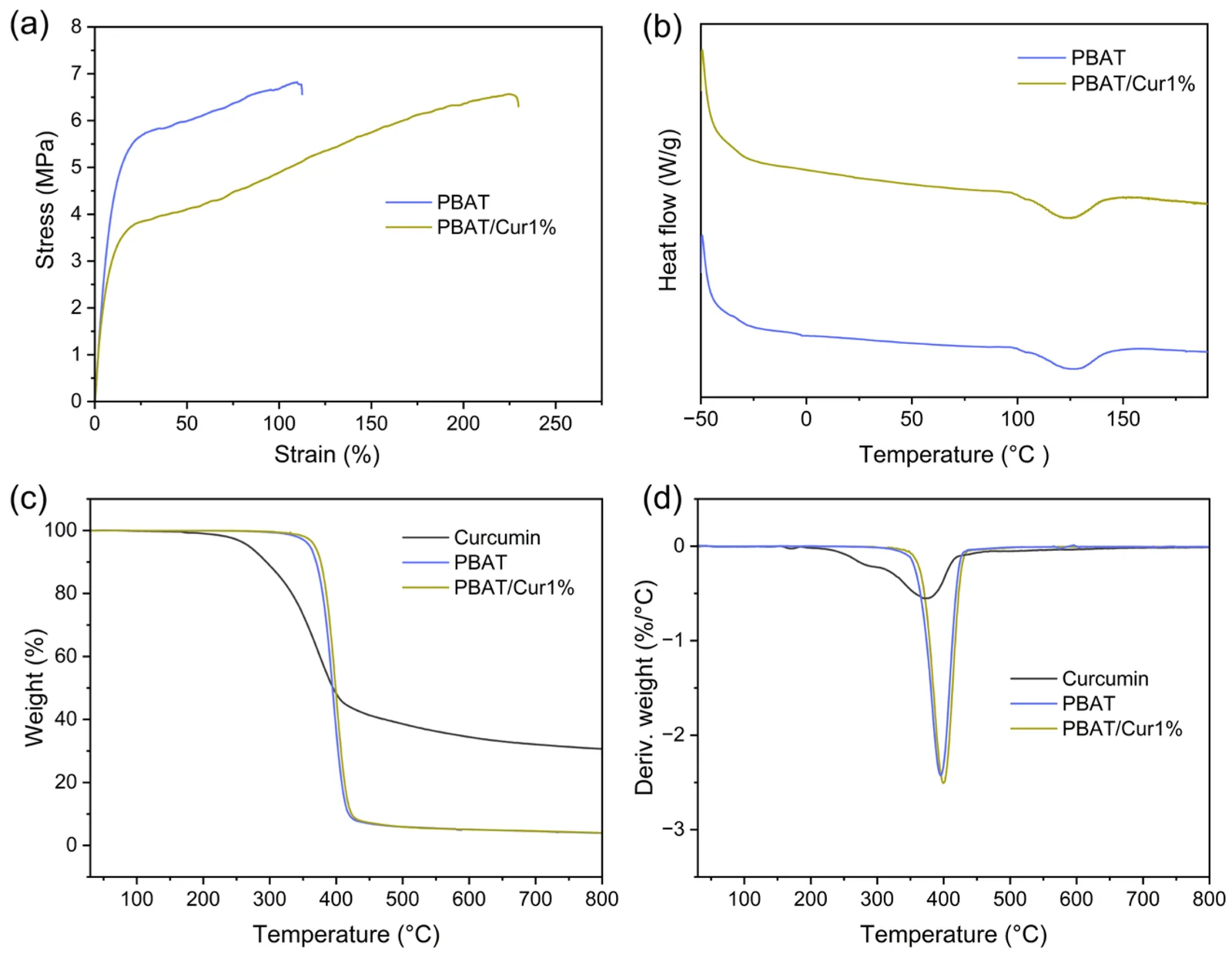
(**a**) Mechanical properties and (**b**) DSC thermograms (second heating cycle) of neat PBAT and PBAT/Cur1% films. (**c**) TGA and (**d**) DTG thermograms of neat PBAT film, curcumin, and PBAT/Cur1% film.

Mechanical properties of the PBAT and PBAT/Cur1% was investigated through tensile tests (Figure 5a). The incorporation of curcumin into the PBAT matrix led to a slight decrease in the tensile strength (6.81 MPa for neat PBAT, and 6.57 MPa for PBAT/Cur1%). However, the elongation at break increased from 110% to 227%. In addition, the elastic modulus decreased moderately from 53.1 to 48.0 MPa. These results indicate that curcumin acts as plasticizer, enhancing chain segmental mobility and the elongation at break.

To further investigate the intermolecular interactions between curcumin and PBAT, DSC analysis was performed on neat PBAT and PBAT/Cur1% films. The melting temperature of PBAT slightly decreased from 126.8 °C to 124.5 °C upon curcumin incorporation (Figure 5b). This modest depression in melting temperature suggests that curcumin molecules partially disrupt the crystalline packing of PBAT chains [47]. This is consistent with the XRD results.

The thermal degradation behavior of neat PBAT, PBAT/Cur films, and curcumin powder under a nitrogen atmosphere is shown in Figure 5c (TGA) and Figure 5d (DTG). Curcumin showed no obvious weight loss below 100 °C, an onset decomposition temperature (T_5_%, defined as the temperature at 5% weight loss) of approximately 270 °C, a maximum degradation rate (*T*_max_) at around 376 °C and a residual char of 30.64% at 800 °C. Neat PBAT film displayed a single-step degradation pattern with a *T*_max_ of ~395 °C, indicating its superior thermal stability compared to curcumin. The incorporation of curcumin did not significantly affect the thermal stability of the composite film, as reflected by the similar degradation behavior.

### 3.8. Barrier Properties

The barrier properties of the PBAT-based films were evaluated in terms of WVP and OTR, both key parameters for food packaging applications. The WVP and OTR of neat PBAT film were determined to be 16.14 g⋅mm/m^2^⋅day⋅kPa and 9.578 × 10^−3^ cm^3^/m^2^⋅d⋅Pa, respectively. Upon incorporation of 1 wt% curcumin, the PBAT/Cur1% film exhibited decreased WVP of 14.58 g⋅mm/m^2^⋅day⋅kPa and OTR of 7.533 × 10^−3^ cm^3^/m^2^⋅d⋅Pa, showing improved barrier properties. This improvement in barrier performance can be ascribed to the uniform dispersion of hydrophobic curcumin in the PBAT matrix, which creates a more tortuous pathway for gas and water vapor molecules, thereby hindering their diffusion through the film [36]. Similar barrier-enhancing effects have also been reported for curcumin incorporated into other polymer matrices, including chitosan [54], cellulose [31,46], PLA/PPC [56], and LDPE [30], where the hydrophobic nature of curcumin contributed to reduced water vapor and oxygen permeability. These results indicate that PBAT/curcumin films exhibit improved barrier properties, which may contribute to their potential application in active food packaging.

Both the neat PBAT film and the PBAT/Cur1% film achieved the maximum kit value of 12, demonstrating no staining even when exposed to the n-heptane/toluene mixture (55/45 vol%). This excellent grease resistance is attributed to the intrinsic hydrophobic nature of PBAT, which effectively resists the penetration of non-polar solvents and oils. Notably, the incorporation of 1% curcumin did not compromise this superior barrier performance.

### 3.9. Antibacterial Activities

**Figure 6:**
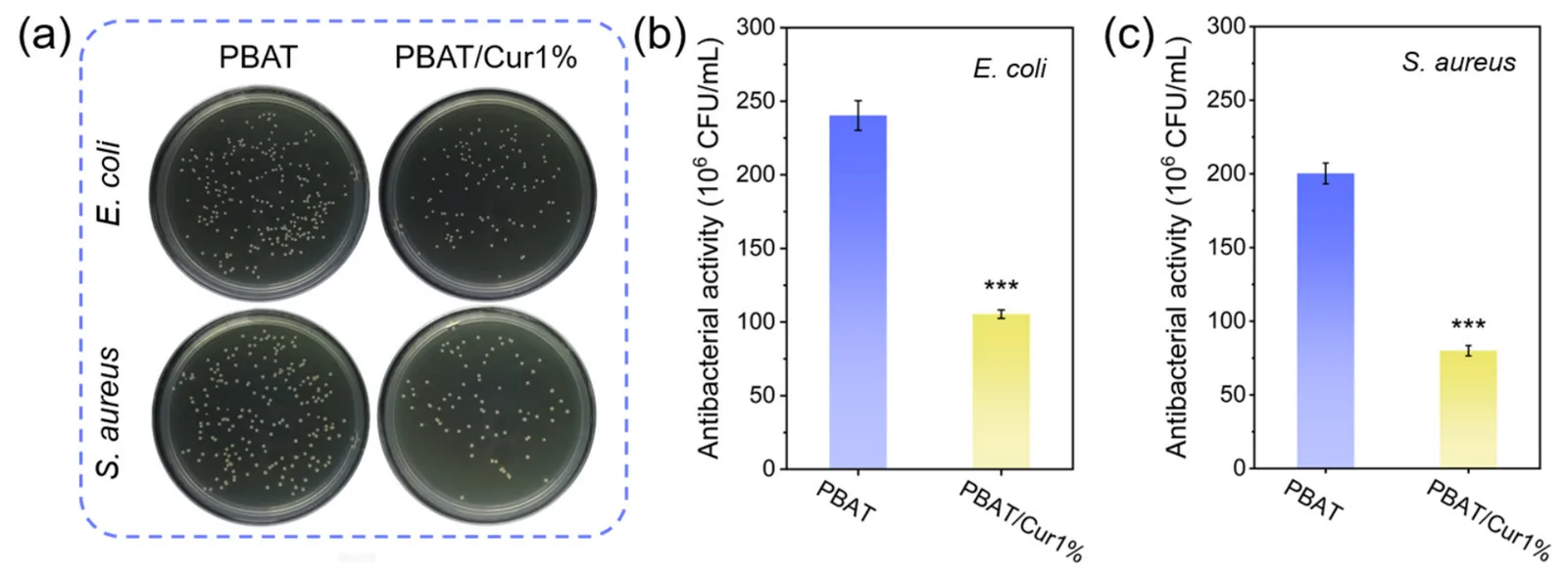
Antibacterial activity of neat PBAT and PBAT/Cur1% films: (**a**) photographs of survived *E. coli* (**top**) and *S. aureus* (**bottom**) colonies on LB-agar plates, and the antibacterial activity against (**b**) *E. coli* and (**c**) *S. aureus*. All values were presented as the mean ± SD (*n* = 3).

Microbial contamination is a major cause of food spoilage during storage, highlighting the need for packaging materials with inherent antibacterial properties for food safety and shelf-life extension. The antibacterial activity of the neat PBAT and PBAT/Cur1% films was tested against *E. coli* and *S. aureus*, using the colony counting method. As shown in Figure 6a–c, the PBAT/Cur1% composite film showed obvious inhibitory effects on both Gram-negative and Gram-positive bacteria, with the number of viable colonies significantly reduced compared to the neat PBAT film (*** *p* < 0.001, independent Student’s *t*-test). The enhanced antibacterial activity is attributed to the incorporation of curcumin, which exerts antibacterial effects through multiple mechanisms, including disruption of bacterial cell membranes, inhibition of DNA replication, and generation of reactive oxygen species [31]. Its amphipathic nature allows curcumin to interact with the lipid bilayer of bacterial membranes, increasing membrane permeability and leading to leakage of intracellular contents [57]. The phenolic hydroxyl groups of curcumin can also interact with the outer lipopolysaccharide layer present in Gram-negative bacteria, weakening the cell wall structure [57]. These results demonstrate that the incorporation of curcumin effectively imparts antibacterial functionality to PBAT films, indicating their promise for active food packaging.

### 3.10. Curcumin Release and Enzymatic Biodegradability

**Figure 7:**
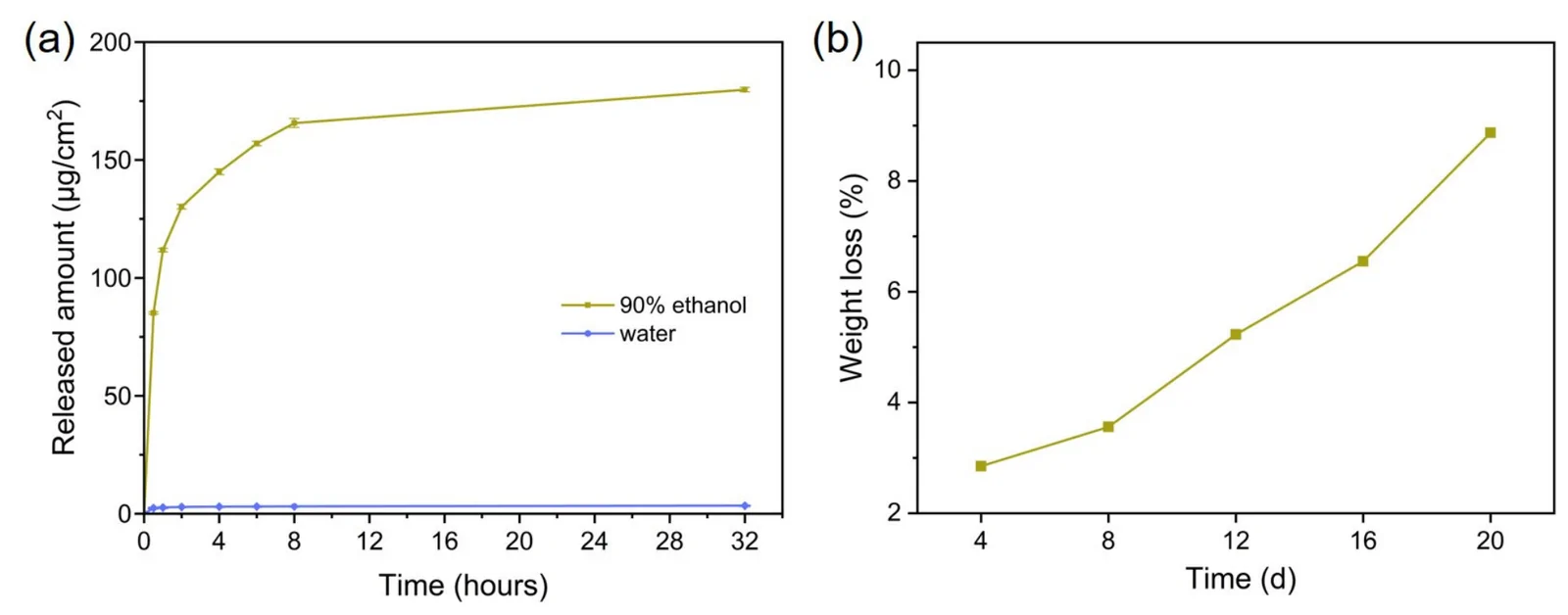
(**a**) The release of curcumin from PBAT/Cur1% and (**b**) enzymatic degradation of the PBAT/Cur1% film.

Curcumin release from the PBAT/Cur1% film was evaluated in water and 90% ethanol as food simulants (Figure 7a). In water, the cumulative release reached only 3.5 μg/cm^2^ after 32 h, attributable to the poor solubility of curcumin and the hydrophobic nature of PBAT, which restricts water penetration. In contrast, a significantly higher release was observed in 90% ethanol (179.8 μg/cm^2^ at 32 h), consistent with the better affinity of curcumin for non-polar media. The release profile in ethanol displayed an initial burst followed by a sustained phase, indicating that surface-bound curcumin was rapidly released, while curcumin embedded in the polymer matrix diffused gradually. The observed release confirms that curcumin is accessible and migrates into fatty food simulants, supporting the antioxidant and antibacterial activities reported in this study. The sustained release behavior is beneficial for long-term active packaging applications. From a safety perspective, curcumin is recognized as a GRAS substance, supporting the suitability of the PBAT/Cur1% film for food-contact applications.

The enzymatic degradation of the PBAT/Cur1% film was evaluated over 20 days in lipase/PBS solution at 45 °C, following a previously reported procedure [58]. As shown in Figure 7b, the mass loss increased progressively with time. After 20 days, the mass loss o reached 8.9%. The degradation is attributed to enzymatic cleavage of ester bonds in the PBAT backbone. The degradation is environmentally favorable, as it reduces the accumulation of non-degradable plastic waste in nature.

### 3.11. Responsive Color-Change Properties

**Figure 8:**
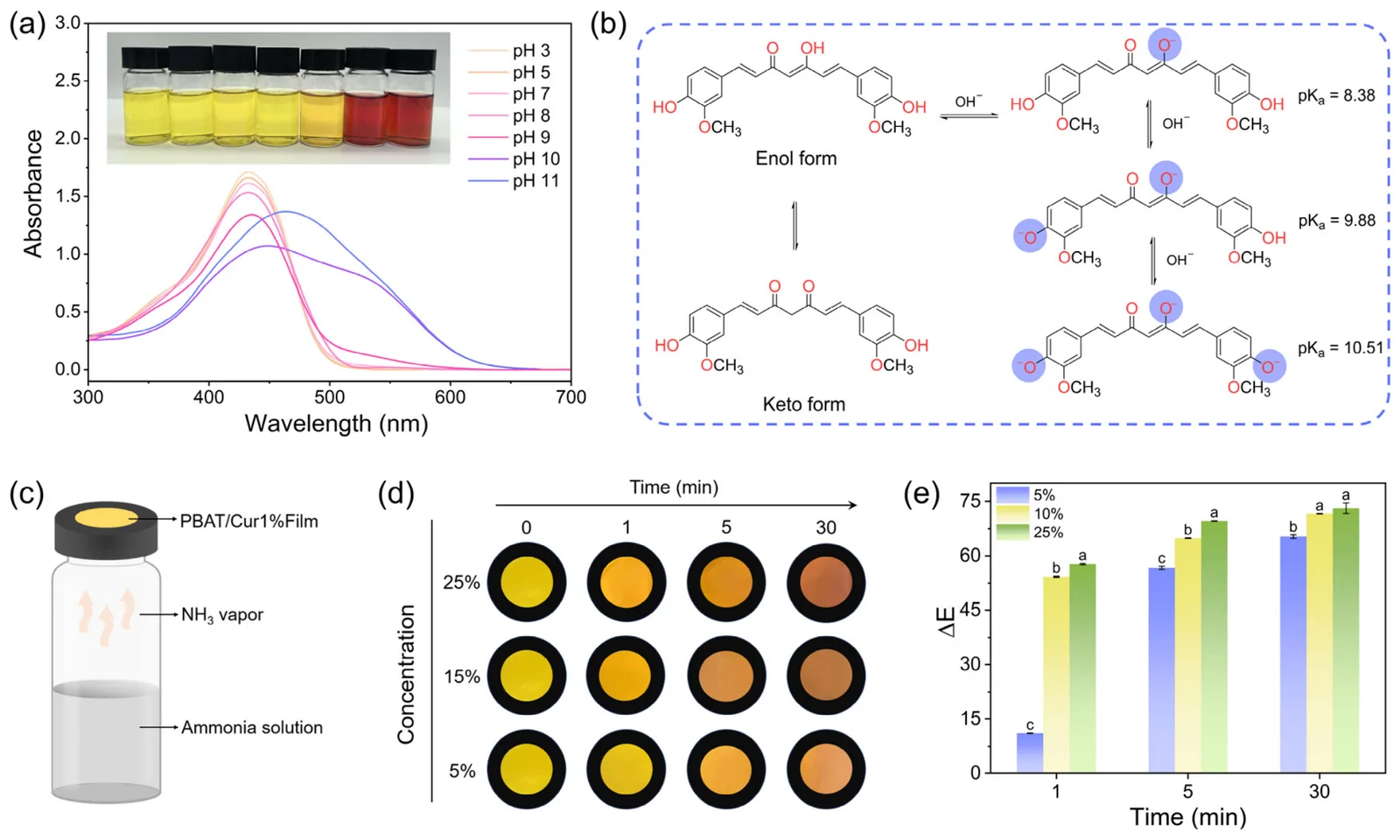
(**a**) UV-Vis spectra of curcumin solutions at pH 3–11. (**b**) Structural changes of curcumin under acidic and alkaline conditions. (**c**) Schematic representation of the colorimetric response of PBAT/Cur1% indicator upon NH_3_ vapor exposure. (**d**) Digital photographs and (**e**) the corresponding color changes of the PBAT/Cur1% indicator upon exposure to NH_3_ vapor generated from ammonia solutions at different concentrations over time. Different lowercase letters indicate statistically significant differences (*p* < 0.05).

The pH-responsive color-changing properties of curcumin were first investigated in solution by UV-vis spectroscopy at different pH values. As shown in Figure 8a, the curcumin solution exhibited a bright yellow color under acidic and neutral conditions, with a characteristic absorption maximum at approximately 435 nm. Upon increasing the pH to 11, the solution color gradually changed from yellow to reddish-brown, accompanied by a red shift of the absorption peak to approximately 465 nm (Figure 8a). This red shift is ascribed to the deprotonation of the phenolic hydroxyl groups in curcumin under alkaline conditions, which extends the conjugated π-electron system and alters the molecular structure (Figure 8b) [31,46]. Such pH-dependent structural transformation provides the molecular basis for the colorimetric response of curcumin-containing materials to alkaline stimuli, including NH_3_ vapor, thereby enabling their application as visual freshness indicators.

Protein-rich foods such as meat and seafood generate alkaline nitrogenous volatiles (e.g., ammonia and dimethylamine) during spoilage [36]. To evaluate the concentration- and time-dependent colorimetric performance of PBAT/Cur1% composite film toward such alkaline volatiles, a vial-based exposure system was established using aqueous ammonia solutions at varying concentrations (5%, 10%, and 25%) to simulate the spoilage-related alkaline atmosphere (Figure 8c). Upon exposure to NH_3_ vapor, the initially yellow films gradually turned reddish-brown, with the color change becoming more pronounced at higher ammonia concentrations and longer exposure times (Figure 8d). The ΔE values increased progressively with increasing NH_3_ concentration and exposure duration (Figure 8e). This colorimetric behavior is consistent with the pH-dependent spectral changes observed in solution (Figure 8a), confirming that the deprotonation of curcumin’s phenolic hydroxyl groups, induced by the alkaline NH_3_ vapor, is the primary mechanism responsible for the color change even when curcumin is embedded in the PBAT matrix. The ΔE larger than 3 can be considered a human perceptible color difference [59]. Furthermore, the color reversibility of the PBAT/Cur1% film was evaluated by cyclic alternation between ammonia and acetic acid vapor. As summarized in Table 2, the film maintained good color-reversible performance over five repeated cycles, demonstrating good reusability. These results demonstrate that the PBAT/Cur1% composite films can serve as effective visual indicators for NH_3_ vapor, with promising potential for real-time monitoring of food freshness in intelligent packaging applications.

## 4. Conclusions

In this study, multifunctional PBAT/curcumin bioactive composite films were successfully fabricated via a simple solution casting method. The incorporation of curcumin effectively imparts potent antioxidant and moderate antibacterial activities to the PBAT matrix, while simultaneously improving its water vapor and oxygen barrier performance. More significantly, the characteristic pH-responsive color transition of curcumin is well retained in the film, enabling a rapid and visually perceptible color change upon exposure to alkaline ammonia vapor, which provides preliminary proof-of-concept for sensing spoilage-related volatile amines. The combination of colorimetric functionality and bioactive properties suggests that the developed films have potential as promising candidate materials for active and intelligent food packaging. Nevertheless, the chloroform-based solution-casting route limits industrial scalability; melt-extrusion, solvent-recovery strategies, or safer solvents should be considered in future work, alongside further evaluation under practical storage conditions to establish their real-world performance.

## Figures and Tables

**Table 1:** Thickness and color parameters of PBAT and PBAT/Cur films.

Sample	Thickness (μm)	L*	a*	b*	ΔE
PBAT	68 ± 1.5 ^d^	89.74 ± 0.11 ^a^	−0.47 ± 0.01 ^c^	−4.61 ± 0.11 ^e^	2.06 ± 0.14 ^e^
PBAT/Cur0.2%	75 ± 3.0 ^c^	86.99 ± 0.04 ^b^	−16.47 ± 0.13 ^e^	72.82 ± 0.30 ^d^	81.00 ± 0.34 ^d^
PBAT/Cur0.5%	82 ± 4.0 ^b^	85.50 ± 0.05 ^c^	−11.02 ± 0.24 ^d^	78.73 ± 0.07 ^c^	86.10 ± 0.11 ^c^
PBAT/Cur1%	91 ± 2.6 ^a^	82.10 ± 0.17 ^d^	0.11 ± 0.08 ^b^	81.27 ± 0.18 ^b^	88.24 ± 0.16 ^b^
PBAT/Cur2%	95 ± 0.6 ^a^	81.16 ± 0.24 ^e^	3.75 ± 0.39 ^a^	85.15 ± 0.37 ^a^	92.29 ± 0.33 ^a^

Different letters in the same column indicate significant difference (*p* < 0.05).

**Table 2:** Reversible color changes of the PBAT/Cur1% film repeatedly exposed to ammonia and acetic acid vapors.

Control	1 Cycle		2 Cycle		3 Cycle		4 Cycle		5 Cycle	
	Ammonia	Acid	Ammonia	Acid	Ammonia	Acid	Ammonia	Acid	Ammonia	Acid
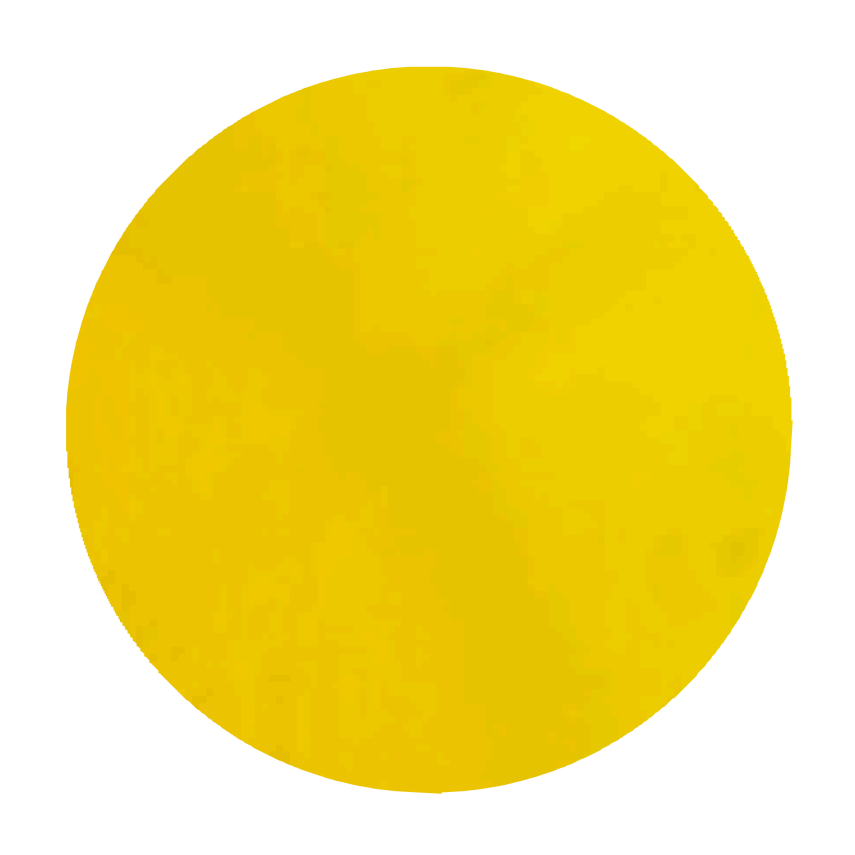	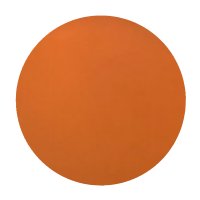	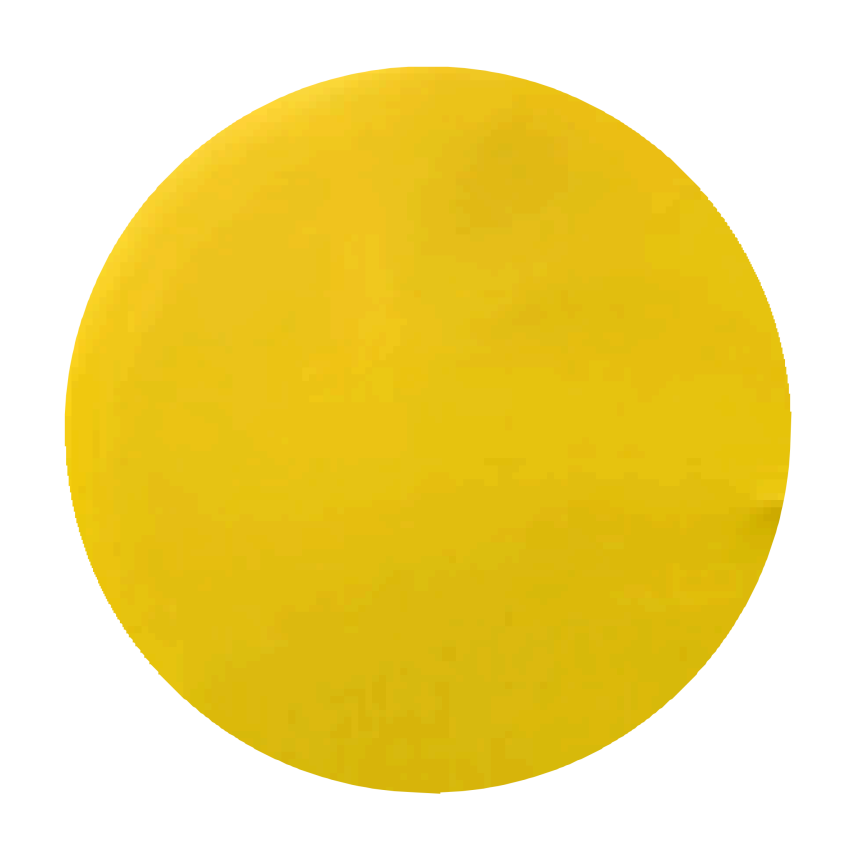	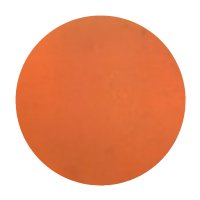	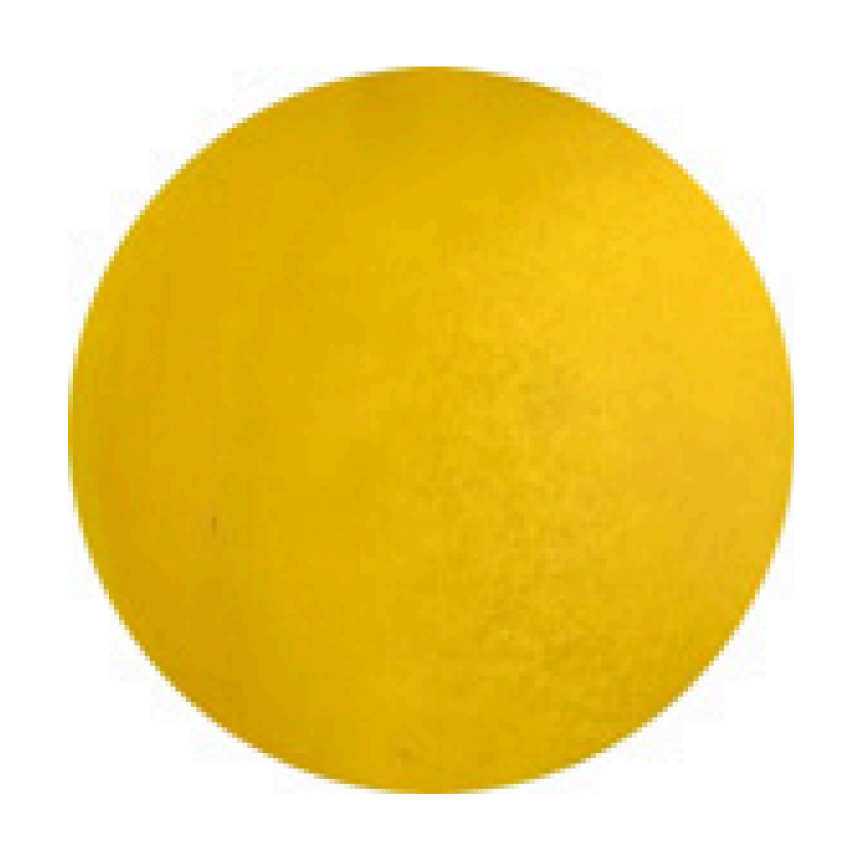	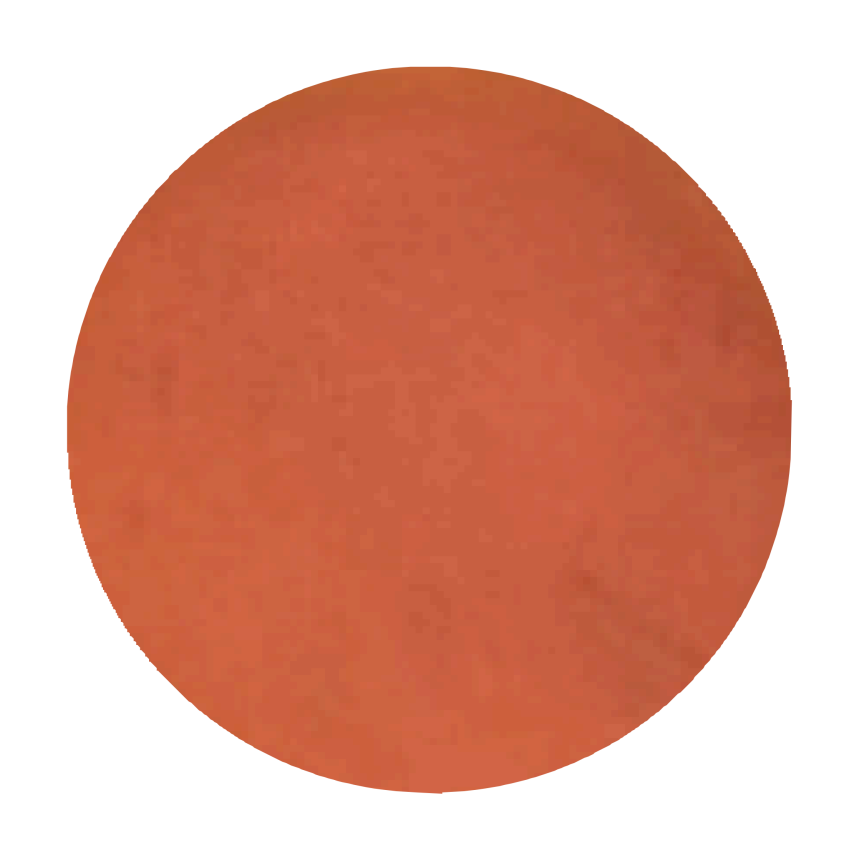	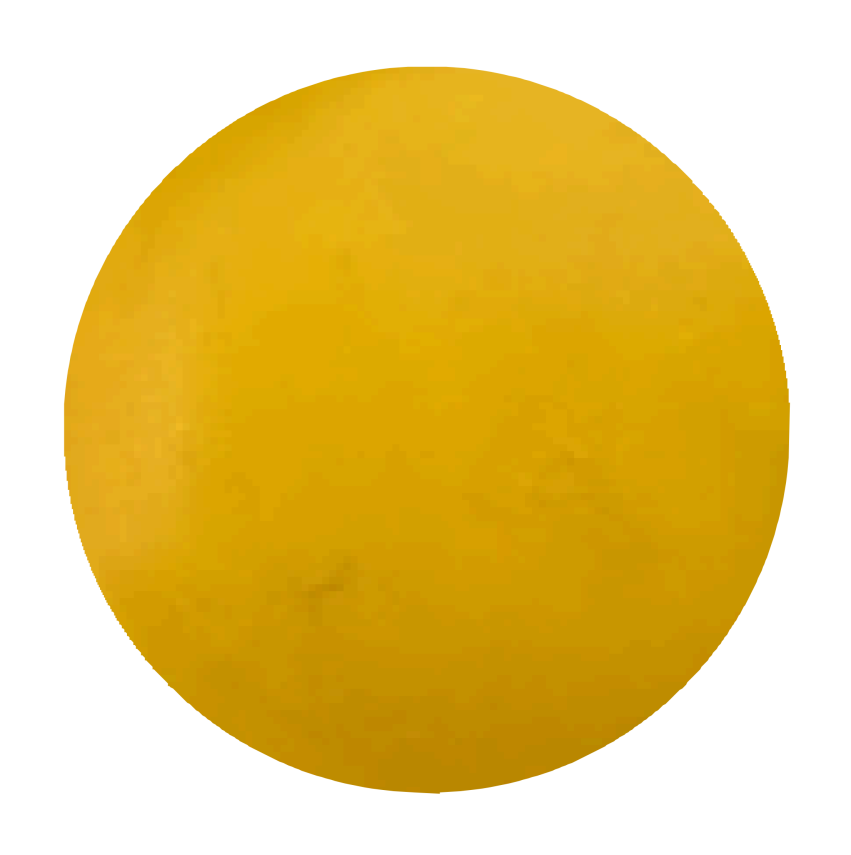	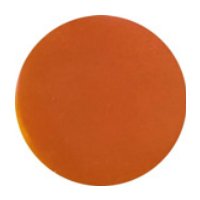	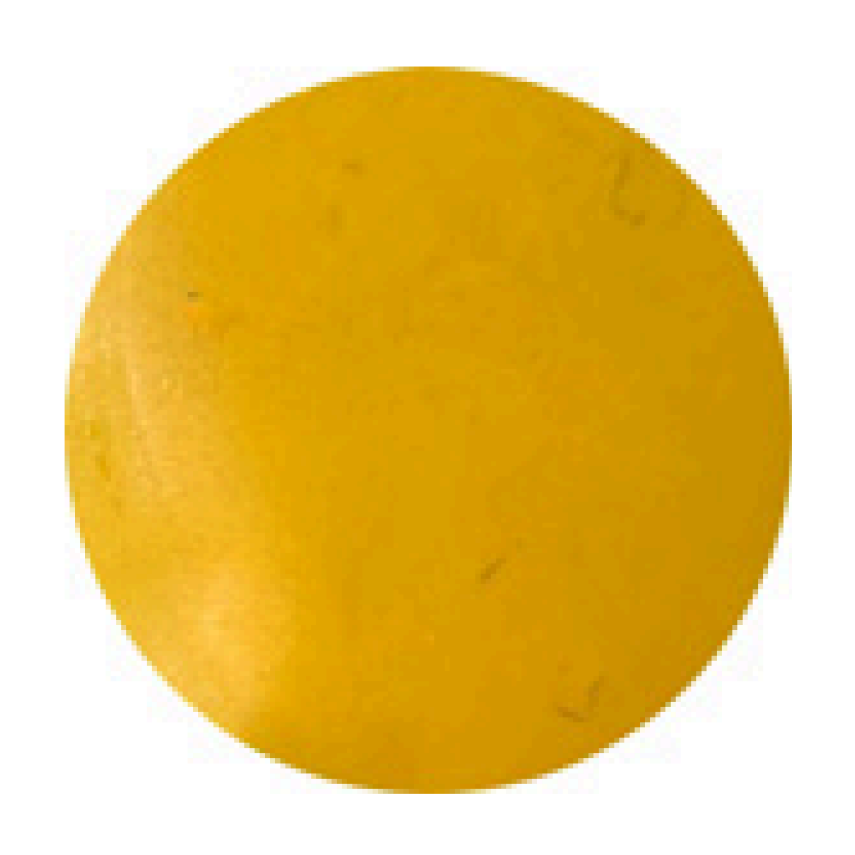	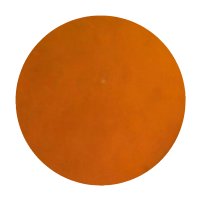	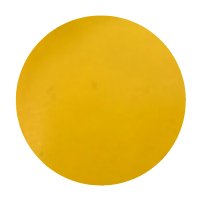

## Data Availability

The original contributions presented in the study are included in the article; further inquiries can be directed to the corresponding authors.
